# Foraging, perennial vegetables, and nutritional resilience in a farmworker community: a mixed-methods analysis comparing nutrition functional diversity and the healthy eating index

**DOI:** 10.3389/fnut.2026.1767089

**Published:** 2026-03-16

**Authors:** Rebecca Garofano, Rick Burnette, Margaret A. Voss

**Affiliations:** 1 Syracuse City School District, Syracuse, NY, United States; 2 Department of Nutrition and Food Studies, Syracuse University, Syracuse, NY, United States; 3 Cultivate Abundance, Fort Myers, FL, United States

**Keywords:** cultural food practices, dietary quality, farmworkers, food security, foraging, nutrition functional diversity, nutrition resilience, perennial vegetables

## Abstract

**Introduction:**

Farmworker communities face significant food access barriers including economic constraints, immigration enforcement threats, and geographic isolation from retail food sources. Foraging practices and perennial vegetable use have been proposed as nutrition resilience strategies in these communities, yet their contributions to dietary quality remain poorly understood. The objective of this study was to compare home garden agrobiodiversity, food access patterns, and dietary quality among farmworker households in Immokalee, Florida, considering foraging and perennial vegetable use as resilience strategies.

**Methods:**

A cross-sectional mixed-methods design was employed, including participatory ranking activities (*n* = 16) and in-depth interviews incorporating dietary recalls and garden mapping (*n* = 58). Data collection was conducted in Spanish and Haitian Kreyòl; Spanish speakers who also spoke an indigenous language were differentiated as a distinct subgroup. Nutrition Functional Diversity (NFD) scores were used to quantify the nutritional contributions of diverse food sources, and Healthy Eating Index (HEI-2015) scores were used to assess overall diet quality. As a cross-sectional study, findings are not temporal and cannot describe causal relationships.

**Results:**

Participants demonstrated diverse food procurement strategies including gardening (67%), foraging (36%), and food pantry use. Mean HEI score was 61.7 ± 12.3, comparable to US population averages. NFD scores revealed that difficult-to-access plants contributed 31.3% of potential nutritional diversity, while pantry-sourced plants contributed 30.1%. Foraged foods contributed notably to micronutrient intake, providing vitamin A (180–200% RDA), calcium (40–60% RDA), and vitamin K. Language group significantly affected HEI scores (*F* = 3.86, *p* = 0.03), with Spanish speakers scoring higher than Haitian Kreyòl speakers. No meaningful correlation was observed between NFD and HEI scores (*R*^2^ = 0.006).

**Discussion:**

The absence of correlation between NFD and HEI scores suggests these metrics capture distinct dimensions of food security and should not be treated as interchangeable. Farmworker communities employ sophisticated nutrition resilience strategies that may not be adequately captured by conventional dietary quality measures, highlighting limitations in applying standardized dietary guidelines across culturally diverse populations. Foraging and perennial vegetable use represent important yet potentially stigmatized food access strategies. Nutrition interventions should recognize and support existing community food knowledge rather than imposing top-down recommendations. Future research should address compositional data gaps for culturally important foods and prioritize development of culturally responsive nutrition assessment tools.

## Graphical Abstract

**Figure:**
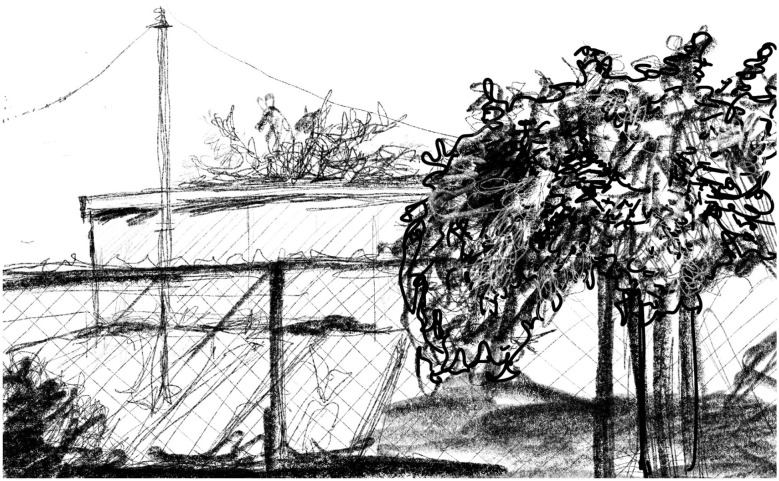
Cover Illustration by Rebecca Garofano: Hoopvine behind Misión Peniel.

## 1 Introduction

Resilience refers to a system's capacity to cope with and recover from shock while addressing vulnerabilities (1). Food systems resilience is a relatively new subject of research and while its importance in protecting food security across both local and national levels is understood (2), little work has been done to specifically consider the nutritional dimensions of this emerging framework. In farmworker communities experiencing challenges through citizenship, migration, labor exploitation, stigmatization, and inadequate access to food, healthcare, and housing (3, 4), nutrition resilience encompasses practices communities employ to address food, health, and nutrition vulnerabilities, withstand food insecurity effects, and recover from its impact.

Immokalee, an unincorporated community in Collier County, Florida (population 26,000), houses 47% of the county's agricultural workforce. Forty-six percent of residents were born outside the United States, primarily in Mexico, Haiti, and Guatemala. The community produces a substantial portion of Florida's tomatoes, which constitute 90% of US winter tomato production (5). Collier County also grows other fruit and vegetable commodities, including peppers, cucumbers, squash, and watermelon. Poverty rates approach three times the national average (6), exacerbated by food costs approximately 25% higher than affluent suburban areas (7). During manuscript preparation, Immokalee's only major grocery store closed (41), underscoring food system vulnerability. Barriers to food access include Immigration and Customs Enforcement (ICE) threats, fear of public charge designation, monolingual service provision, and transportation limitations (L. Vazquez Reyes, personal communication, May 13, 2022). Immigration raids intensified in 2025 (8), restricting wage-earning ability and destabilizing family and neighborhood economies.

Farmworkers remain historically overlooked in emergency food operations, not only as producers and laborers but as consumers with unique culture, knowledge, and skills (9). This exclusion, whether inadvertent or deliberate, can occur through emergency food projects intended to support these communities (3). Chronic sociocultural stressors shaped by poverty and historic oppression produce trauma (10, 11). This chronic stress reflects what Scheper-Hughes and Bourgois term structural, symbolic, and everyday violence experienced by marginalized populations (12). Holmes uses “everyday violence” to capture commonplace micro-expressions of violence and humiliation in Mexican farmworkers' experiences (44). Persistent stress contributes to chronic disease risk through metabolic pathways and reduced cognitive bandwidth and resilience (13). In Immokalee, trauma manifests both in specific ICE raids separating families without due process (14) and in grocery store closures that force hour-long travel to more expensive alternatives.

The nutrition resilience framework recognizes the context farmworker communities navigate and their utilization of unique knowledge, experiences, and preferences to access food. Evidence of these methods appears in subtle ways: potted herbs on trailer doorsteps, papaya exchanges with neighbors, selection of young vine tips extending over fences, or use of perennial vegetables within the broader ecosystem. When emergency food pantries, support organizations, community nutrition programs, and allied health professionals recognize these nutrition resilience tools, the plants used, and their meaning to people, opportunities arise to practice cultural humility—the foundation of responsive and meaningful care.

This research explores how Immokalee community members access foods from the landscape through foraging perennial vegetables as one nutrition resilience strategy. The objective of this study was to compare the agrobiodiversity of home gardens to the dietary patterns of community members in Immokalee, Florida. The hypothesis was that greater garden diversity resulted in greater diet diversity. With a mixed methods approach, it uniquely employs an ecological framework via nutrition functional diversity scores alongside recommended diet patterns via a healthy eating index score.

## 2 Materials and methods

### 2.1 Recruitment and participants

Participants were recruited via snowball sampling by research facilitators: Lupita Vazquez (Cultivate Abundance staff) and Helen Midney (community partner gardener) facilitated Spanish activities, while Frantzso Marcelin (contract worker) facilitated Haitian Kreyòl activities. Initial recruitment occurred through word-of-mouth at Misión Peniel food pantry distributions and the Cultivate Abundance garden. Participants who attended participatory ranking activities were invited to individual interviews and recommended friends or neighbors. Frantzso additionally recruited Haitian Kreyòl speakers at Immokalee's weekly open-air market.

Participatory Ranking Activity (PRA) inclusion criteria required adult Immokalee residents. Interview inclusion criteria specified women living in Immokalee (any residence duration, occupation, migration status, or housing situation). Men and individuals under 18 were excluded. Participants received $20 (PRA) or $15 (interview) Family Dollar gift cards. The initial target of 10 PRA participants and 30 interviews was exceeded: 16 participated in PRA (11 Spanish speakers, 5 Haitian Kreyòl speakers), and 58 interviews were completed with additional Southwest Regional Planning Committee funding. While the sample size was small due to limited resources, recruitment was focused on individuals navigating constrained food resources and is adequately representative of those attending the Misiòn Peniel food pantry.

### 2.2 Participatory ranking activities

PRA methodology involves group rapid response to questions, building consensus and creating comprehensive topic understanding (43). Rick Burnette (Cultivate Abundance) facilitated the 1-h session with Lupita, Helen, and Frantzso support. Participants received snacks, refreshments, gift cards, and produce bundles. The session sought participant perspectives on defining and ranking culturally important food plants by preference, accessibility difficulty, and acquisition source (pantry, store, garden, exchange, or foraging). Language groups were specified as Haitian Kreyòl and Spanish (Indigenous language speakers were not differentiated).

**Figure 1:**
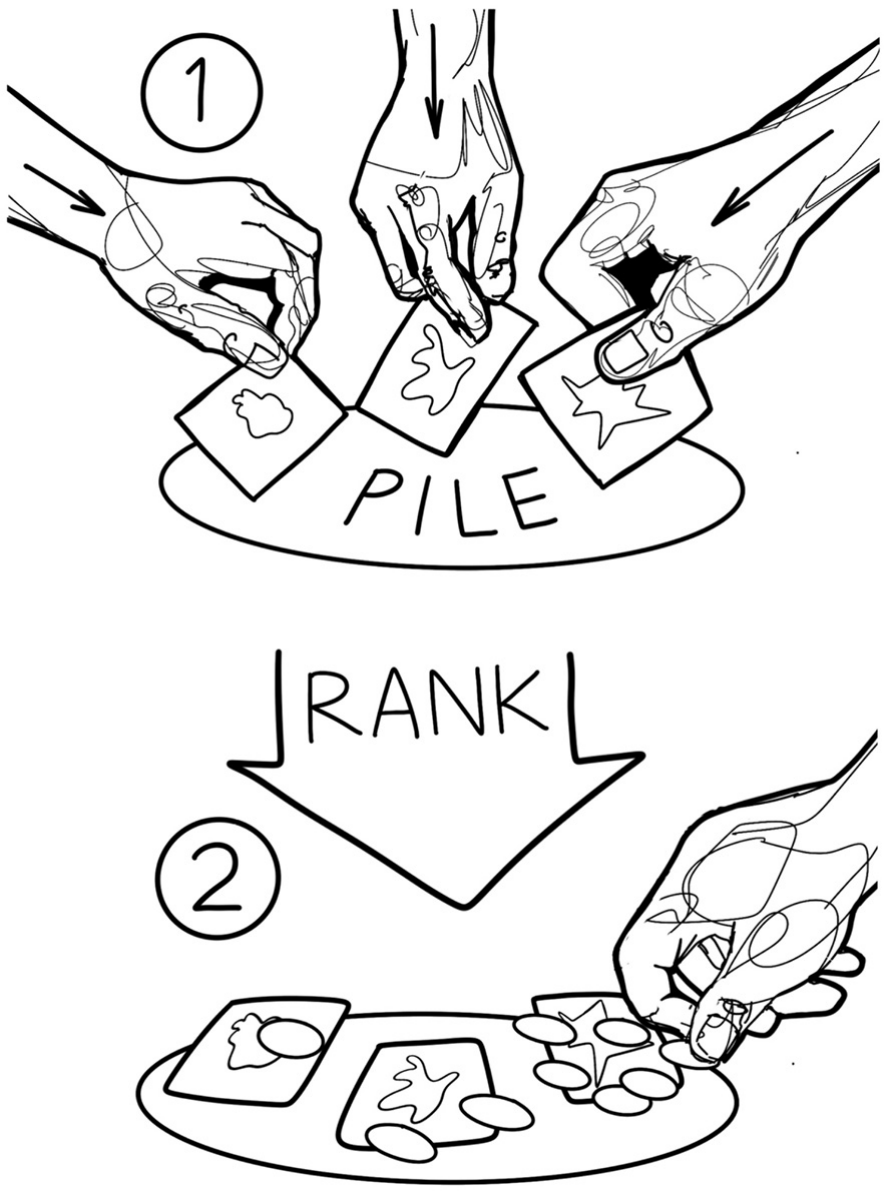
Visualizing the participatory ranking activity (PRA), a two-step process of (1) collecting contributions from group members and (2) voting on which contributions are the most pertinent. Illustration by Rebecca Garofano.

The garden-based session split participants into language groups for two activities (Figure 1). First, a “piling” activity asked participants to list preferred fruits or vegetables (excluding grains and dry pulses). Participants used five sticky notes to write the most culturally important plants, phrased as “as a ______ (Haitian, Guatemalan, or Mexican) person living in Immokalee, the most important food to me is ______.” Groups shared and compiled lists. Second, a “ranking” activity involved voting on most important plants, then voting on most difficult to access plants. Subsequent activities ranked plants by source (pantry, garden, foraging, store, or exchange). Plant lists generated nutrition composition matrices.

### 2.3 Individual interviews: diet recall and garden maps

Fifty-eight women provided informed consent and received unique IDs; all analysis used deidentified data. Spanish and Haitian Kreyòl interviews collected demographic data including language, housing type (trailer, house, rented room, rent/own status), age, and US residence duration. Each participant completed a 24-h diet recall and garden map exercise. Cultivate Abundance staff selected recalls over food frequency questionnaires for efficiency and relational informality. Portion sizes used hand measurements (e.g., fist, thumb). Home-cooked meal descriptions included complete preparation details. As with diet recall data, it is possible that this data was impacted by measurement error and social desirability bias, resulting in inflated HEI scores and diet diversity scores. The methodology would have been strengthened by a larger sample size. Diet recall information was entered into the ESHA Food Processor Database (20). Whenever possible, USDA SR Legacy or USDA FNDDS data was used. ESHA Food Processor reports were produced for the complete 24 recalls, with 64 individual nutrients reported. ESHA MyPlate reports were also produced for each interviewee. Garden maps used consistent templates listing all food plants around living areas. Grid patterns indicated relative space occupied by each plant. The recall method used to evaluate gardens was potentially impacted by recall bias and that assessing garden spaces in person would have likely increased garden diversity scores.

### 2.4 Nutrition functional diversity assessment

This study applies nutrition functional diversity (NFD) metrics to assess nutritional resilience. NFD adapts ecological frameworks to nutrition science by evaluating how diverse food sources contribute to meeting nutritional needs. Drawing from functional diversity (FD) metrics in ecology using trait-based biodiversity quantification approaches (15), NFD assesses plant species range and interactions within food environments (16). NFD is defined as “distinct species in a population that have unique functional traits” (17).

NFD frameworks prove valuable for understanding nutrition resilience by capturing both food species variety available to communities and the nutrient assemblage or nutritional functional groups these species provide. Evaluating participants' growing spaces, foraging practices, and food access patterns through NFD identifies synergies and tradeoffs between agricultural practices and nutritional outcomes (18, 19). This approach examines how different food sources collectively support dietary adequacy and community food security beyond simple species counting, revealing nutrition resilience patterns. We use a definition of nutrition resilience that focuses on the capacity of a food system to provide adequate nutrition even when facing environmental, economic, or social challenges.

Following the methodology of Luckett et al. (19) for Malawian households, we created a large matrix with three subcomponents: (1) PRA plant composition matrix, (2) garden map composition matrix, and (3) 24-h diet recall composition matrix. Each matrix row represented an identified food item; each column represented a nutrient. All nutrition composition data was reported per 100 g. The PRA session identified 83 food plants (each a unique row), weighted by relative votes adjusted for language group participant numbers. The 58 interviews identified 98 garden plant species, weighted by occupied space. Following Luckett et al. (19), diet recall excluded processed foods (e.g., alcohol, caffeine, salt, restaurant items), leaving 160 items weighted by consumption amount. The final combined matrix included 242 unique food items.

Nutrition composition values came from ESHA Research Food Processor database (20). Wherever possible, United States Department of Agriculture (USDA) National Nutrition Database for Standard Reference Legacy (SR Legacy) or Food and Nutrient Database for Dietary Studies (FNDDS) were used from within the ESHA database. Where plant composition data was not available, the United Nations International Network of Food Data Systems (INFOODS) was referenced, as well as Toensmeier et al.'s “Perennial Vegetables: A Neglected Resource of Biodiversity, Carbon Sequestration, and Nutrition” (21). The composition matrix included 34 nutrient values.

The next step required converting the nutrient matrix into a food-food distance matrix where each row and column represented a plant species and each cell represented the Euclidean distance between species, again following the methodology of Luckett et al. (19). The food-food matrix was subsequently used for a cluster analysis grouping foods by nutrient similarities and distances between/within food clusters using an unweighted pair group method. Step four calculated dendrogram branch length distances. The highest potential NFD score summed all 242 plant branch lengths. Individual NFD scores were calculated as: (individual household NFD)/(total potential NFD) × 100 (19). FDiversity software (22) calculated all scores. Home garden and individual NFD scores were calculated for PRA activities and individual diet recalls, expressed as percentage of total NFD available to each participant group.

Spider graphs of 400-g samples of the different plant lists were created based on Tong et al.'s meta-analysis evaluating fruit and vegetable consumption for a healthy heart (23). These graphs consider the ways that different food access points provide different micronutrients, and were compared to the US Food and Drug Administration's recommended intake.

### 2.5 Healthy eating index assessment

Individual Healthy Eating Index (HEI) scores followed USDA Center for Nutrition Policy and Promotion HEI-2015 components and scoring standards. The HEI comprises thirteen subscores: nine adequacy scores and four moderation scores (24–26). Maximum score is 100 points.

### 2.6 Statistical analysis

Statistical analyses examined relationships between demographic factors, food access patterns, diet quality (HEI), and nutrition resilience (NFD). Data distributions were assessed for normality using appropriate parametric or non-parametric tests. Levene's Test verified equal variance assumptions.

For PRA components, NFD scores were calculated for each ranking activity for the full group and by language group (Spanish, Haitian Kreyòl). One-way between-groups ANOVA explored language impact on average NFD scores, with language serving as a proxy for how culture shapes food patterns, preferences, and practices.

HEI scores from 58 interviews were normally distributed, permitting parametric analysis. Independent samples *t*-test explored garden ownership impact on HEI scores. Two-way between-groups ANOVAs explored housing conditions (house, trailer, rented room) and language group (Haitian Kreyòl, Spanish, Spanish+Indigenous language) impacts on HEI scores. *Post-hoc* Tukey's HSD tests identified specific language group differences.

Chi-square independence tests explored relationships between language and foraging/gardening patterns. Direct logistic regression assessed combined housing and language impacts on foraging likelihood. Three-way between-groups ANOVA evaluated language group, foraging behavior, and housing condition interactions on HEI scores. Independent samples *t*-tests explored foraging impacts on diet NFD and HEI scores. IBM SPSS Statistics 28 was used to conduct all analyses (α = 0.05) (39).

## 3 Results

### 3.1 Participant demographics

Table 1 presents demographic data. Mean participant age was 51 ± 15.25 years (median 52). US residence duration ranged from 0.75 to 65 years, with a mean of 17.89 ± 13.02 years (positively skewed, median 14.5). Of 58 women interviewed, 39 maintained gardens (small containers to fruit orchards). Twenty-one individuals reported foraging within the prior month. Mean caloric intake was 1,448 ± 697 kcal (median 1,309), water intake 1.3 ± 0.6 L (median 1.2), sodium intake 2,474 ± 2,071 mg (median 1,987). HEI scores ranged 34.92–87.53, mean 61.73 ± 12.27 (median 62.68), comparable to the US population average of 58 (26).

**Table 1:** Demographics and descriptive statistics for surveyed food pantry participants in Immokalee, Florida.

**Variable**	***N***	**Mean**	**Median**	**SD**	**Min**	**Max**
Age	42	50.88	52.00	15.25	26.00	76.00
Years in the US	42	17.89	14.50	13.02	0.75	65.00
HEI score	58	61.72	62.68	12.27	34.92	87.53
Caloric intake (kcal)	58	1,447.77	1,308.88	697.08	301.84	4,121.14
Water (g)	58	1,300.00	1,199.36	611.92	300.32	3,864.03
Sodium (mg)	58	2,473.63	1,986.63	2,070.75	336.09	11,549.10
Diet NFD score	58	8.32%	8.75	4.43	0.32	17.22
Garden NFD score	58	2.62%	2.76	1.340	0.10	5.42

During PRA, 83 food plants were listed and ranked, 17 foraged. Each plant species was weighted by votes received, standardized between five Haitian Kreyòl and eleven Spanish speakers. The 58 interviews identified 98 home garden plant species. Twenty-two of 58 interviewees reported past-month foraging, listing 18 plant species. To determine NFD of growing spaces, plant species were weighted by the relative garden map grid space, which was represented by a grid. The weighted scores were also adjusted for home vs. trailer plot size differences.

### 3.2 Participatory ranking activity results

Table 2 presents NFD scores for each PRA ranking activity, for both full group and by language group. NFD scores reflected potential diversity of 34 nutrient values in specific plant assemblages identified through each activity (preferred plants, difficult-to-access plants, various source plants). Plants listed as difficult to access produced the highest total NFD percentage (31.3%), just above plants accessed from Misión Peniel food pantry (30.1%). Foraged plants had the lowest overall NFD score (17.4%).

**Table 2:** Nutrition functional diversity (NFD) scores from the participatory ranking activity (PRA) for all participants.

**Data source**	**Raw functional diversity score**	**%NFD all PRA plants (83)**	**%NFD diet recall plants (160)**	**%NFD all plants in data set (241)**
All plants from diet recalls (160)	1,798.18		100.00	
All PRA plants (83)	546.18	100.00	30.37	0.27
Culturally preferred	162.86	29.80	9.06	0.08
Access	171.02	31.30	9.51	0.08
Garden	113.88	20.90	6.33	0.06
Pantry	164.64	30.10	9.16	0.08
Forage	95.19	17.40	5.29	0.05
Store	158.36	29.00	8.81	0.08
Exchange	100.12	18.30	5.57	0.05
All plants in dataset (241)	203,618.24			100.00

The %NFD represents the NFD score for plants identified in each activity (PRA or diet recall) expressed as a percentage of the total potential NFD score across all possible plants reported. For example, the plants participants identified as provided by the pantry during the diet recall achieved an NFD score representing only 9.16% of the total potential nutritional diversity available across all plants used by all participants. This indicates that while individuals may prefer a wide array of nutritionally diverse plant species, the subset of plants a given individual might actually be able to access provided a relatively narrow portion of the total nutritional potential.

Table 3 shows NFD scores for separate language groups. Haitian Kreyòl speakers reported highest potential NFD percentage from store-purchased plants (19.77%), then pantry-accessed plants (17.89%). Spanish speakers had highest NFD scores from pantry plants (20.28%), then store-purchased plants (19.42%). Spanish speakers' NFD scores for garden-accessed and exchanged plants exceeded Haitian Kreyòl speakers (19.61% vs. 4.50%; 17.73% vs. 0.95%, respectively). Haitian Kreyòl PRA participants listed no foraged plants.

**Table 3:** Nutrition functional diversity (NFD) scores from participatory ranking activity (PRA) and diet recall, by language group.

**Language**	**Data source**	**Functional diversity score**	**%NFD all plants used within a language**	**%NFD PRA plants (83)**	**%NFD diet recall plants (160)**
Haitian Kreyòl	All plants reported	394.64	100.00	72.25	21.95
	Culturally preferred	88.29	22.37	16.17	4.91
	Access	89.16	22.59	16.32	4.96
	Garden	24.56	6.22	4.50	1.37
	Pantry	97.73	24.76	17.89	5.43
	Store	107.98	27.36	19.77	6.00
	Exchange	5.20	1.32	0.95	0.29
Spanish	All plants reported	244.72	100.00	44.81	13.61
	Culturally preferred	113.67	46.45	20.81	6.32
	Access	96.89	39.59	17.74	5.39
	Garden	107.13	43.78	19.61	5.96
	Pantry	110.74	45.25	20.28	6.16
	Forage	96.00	39.23	17.58	5.34
	Store	106.07	43.34	19.42	5.90
	Exchange	96.85	39.58	17.73	5.39

The %NFD represents the NFD score for plants identified in each activity (PRA or diet recall) expressed as a percentage of the total potential NFD score across all possible plants reported by that language group. For example, the plants Haitian Kreyòl speakers reported as received from the pantry during the diet recall achieved an NFD score representing only 5.43% of the total nutritional diversity available across all plants potentially used by Kreyòl-speaking participants. This indicates that while individuals may prefer a wide array of nutritionally diverse plant species, the subset of plants available provided a relatively narrow portion of the total nutritional potential

Language likely serves as a proxy for how culture shapes food patterns, preferences, and practices. One-way between-groups ANOVA explored language impact on average NFD scores, revealing significant differences in main NFD score effect for language groups [*F*(2, 15) = 3.62, *p* = 0.05]. Spanish-speaking groups had higher average functional diversity scores with less variability.

**Figure 2:**
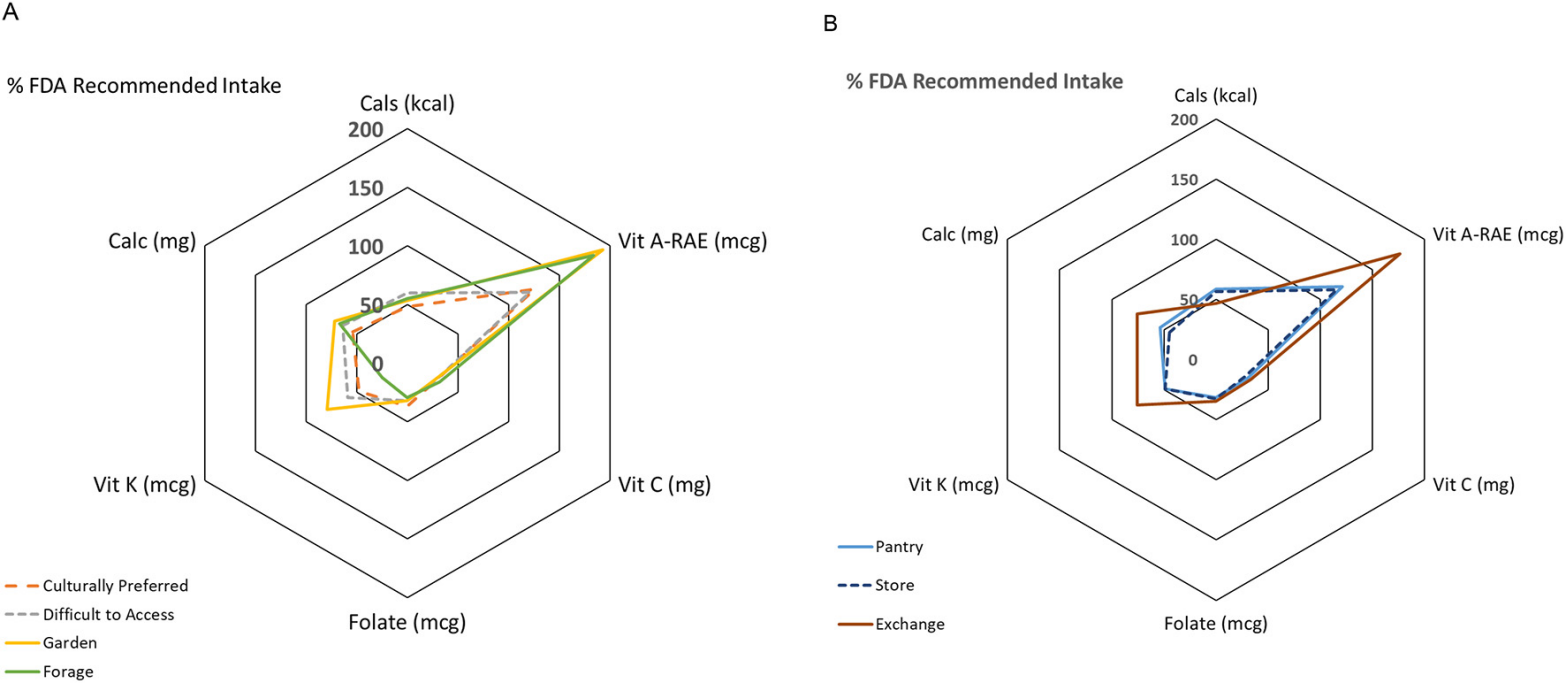
Percentage of select micronutrients from a composite nutrient analysis of 400g of plant foods as compared to the US FDA recommended daily intake. **(A)** Plant foods that are preferred, difficult to access, foraged, or gardened. **(B)** Plant foods that are routinely accessed by food pantry participants through pantry, store purchase, and exchange.

PRA plant lists were used to generate spider graphs of 400 g samples comparing micronutrient provision from different food access points against US FDA recommended daily intake (Figure 2). Plants listed as most commonly gardened, foraged, and exchanged provided highest Vitamin A-RAE levels (180%−200% daily recommended intake) and higher calcium levels (40%−60% RDA) compared to pantry and store plants (20%−40%). Gardened and exchanged plants provided relatively higher Vitamin K levels (approximately 60% FDA daily intake).

### 3.3 Interview and garden results

HEI scores from 58 interviews were normally distributed. Levene's Test verified equal variances. Average participant subscores were notably high for total vegetables (mean 3.93 ± 1.56/5.00, median 5.00), total protein (mean 4.26 ± 1.51/5.00, median 5.00), and refined grains (moderation subscore: mean 8.66 ± 2.63/10.00, median 10.00). Lower scores appeared for dairy (mean 2.56 ± 3.48/5.00, median 0.48), sodium (mean 3.06 ± 4.46/10.00, median 0.00), and whole fruit (mean 1.60 ± 2.33/5.00, median 0.00).

A *T*-test exploring garden impact on HEI scores showed no statistically significant effect [*F*(1, 56) = 1.93, *p* = 0.17]. Two-way between-groups ANOVA exploring housing condition (i.e., house, trailer, rented room) impact on HEI scores showed no statistically significant effect [*F*(2, 40) = 1.41, *p* = 0.26].

Two-way between-groups ANOVA was used to explore language impact on HEI scores. Interview participants were categorized into three language groups: Haitian Kreyòl, Spanish exclusively, and Spanish+Indigenous language (representing eight different indigenous languages). Significant differences appeared in mean HEI scores across language groups (*F*(2, 55) = 3.86, *p* = 0.03). *Post-hoc* Tukey HSD comparisons indicated the Kreyòl-speaking group mean HEI score (*M* = 56.59, SD = 10.32) differed significantly from the Spanish-speaking group (*M* = 65.99, SD = 10.80). The Spanish+Indigenous language group (*M* = 64.06, SD = 14.16) did not differ significantly from other groups (Table 4). This higher HEI score within the Spanish speaking group means that, on average, the Spanish speaking participants had a significantly higher diet quality than participants that spoke Haitian Kreyòl, as measured by the thirteen subscores outlined above in the methodology section. This result may have been an artifact of an unquantified increased access to land by Spanish speaking participants, although our research methods do not permit clarification of that point.

**Table 4:** Results of three-way ANOVA examining the effects of foraging behaviors, housing, and language on HEI scores.

**Source**	**Type III sum of Sq**	**df**	**Mean Sq**	**F**	**Sig**.	**Partial Eta squared (η^2^*p*)**
Corrected model	2,598.876^a^	12	216.57	1.62	0.14	0.39
Intercept	87,468.80	1	87,468.80	656.14	0.00	0.96
Language	1,193.23	2	596.62	4.48	0.02	0.23
Forage	3.88	1	3.88	0.03	0.87	0.00
Housing	949.83	2	474.91	3.56	0.04	0.19
Language ^*^ Forage	503.19	1	503.19	3.77	0.06	0.11
Language ^*^ Housing	309.69	2	154.85	1.16	0.33	0.07
Forage ^*^ Housing	465.12	1	465.12	3.49	0.07	0.10
Language ^*^ Forage ^*^ Housing	7.40	1	7.40	0.06	0.82	0.00
Error	3,999.26	30	133.31			
Total	175,548.95	43				
Corrected total	6,598.13	42				

^a^*R* squared = 0.394 (Adjusted R squared = 0.151).

### 3.4 Food access patterns by language group

Chi-square independence tests explored language and food access pattern relationships. Gardening was significantly associated with language [χ^2^(2, 58) = 33.315, *p* < 0.001]: 100% of Spanish speakers, 93.8% of Spanish+Indigenous speakers, and 25% of Haitian Kreyòl speakers reported home gardens. Conversely, foraging showed no significant language group association [χ^2^(2, 58) = 4.612, *p* = 0.10], despite higher prevalence among Haitian Kreyòl speakers (54.2%) vs. Spanish speakers (27.8%) and Spanish+Indigenous speakers (37.9%). Logistic regression including language and housing as foraging behavior predictors was not statistically significant.

### 3.5 Foraging impact on diet quality

**Figure 3:**
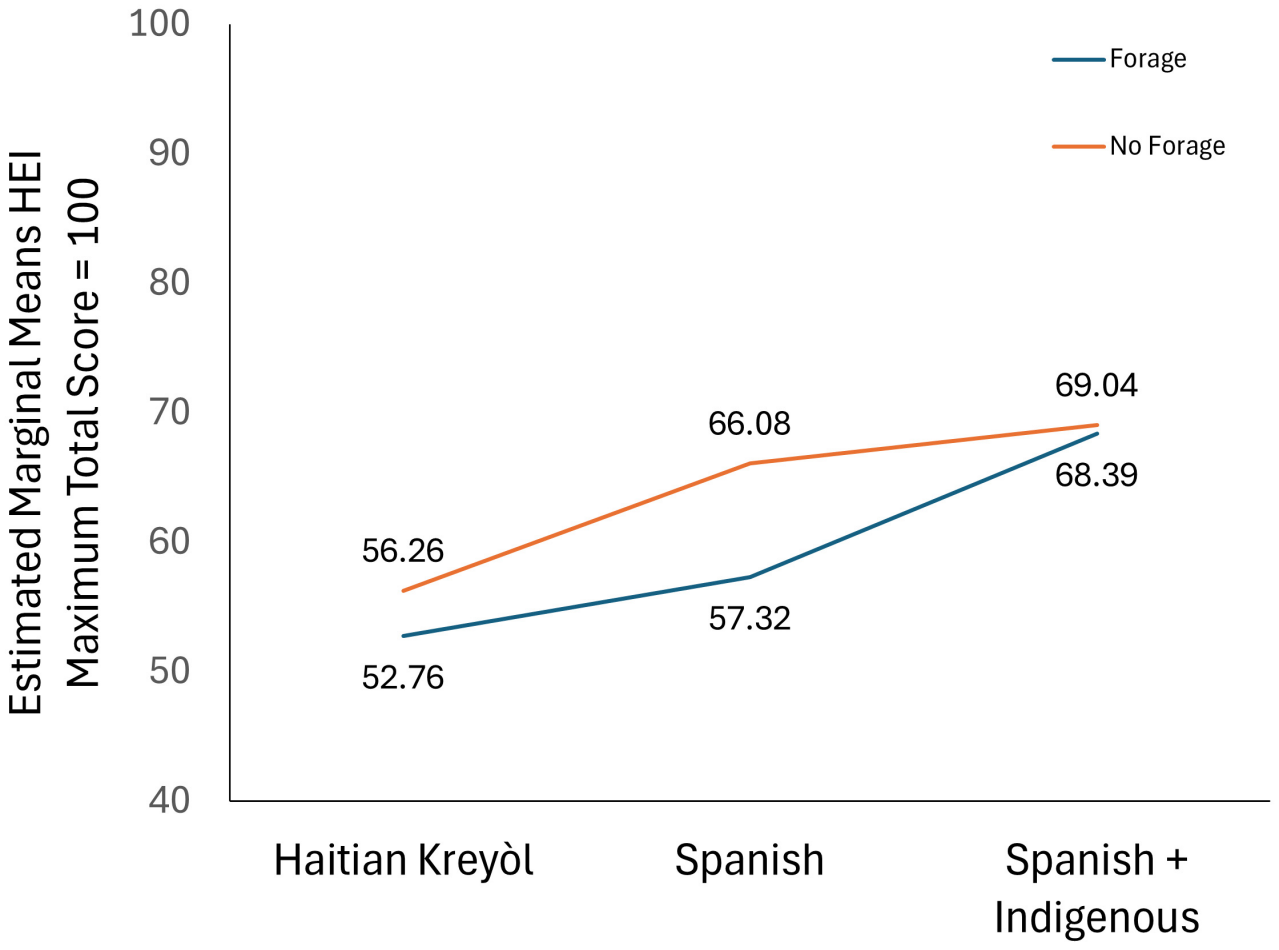
The effect of the interactions between foraging and language on HEI score. Participants that reported the use of foraging exhibited lower HEI scores.

Three-way between-groups ANOVA evaluated language group, foraging behavior, and housing condition interactions on HEI scores (Figure 3). The three-way interaction was not statistically significant [*F*(1, 30) = 0.06, *p* = 0.82]. However, significant main effects emerged for language [*F*(2, 30) = 4.48, *p* = 0.02] and housing [*F*(2, 30) = 3.56, *p* = 0.04]. Language-foraging interaction approached significance [*F*(1, 30) = 3.77, *p* = 0.06], as did foraging-housing interaction [*F*(1, 30) = 3.49, *p* = 0.07]. The main effect of foraging, on its own, was not significant [*F*(1, 30) = 0.03, *p* = 0.87].

Independent samples *t*-tests revealed non-foraging participants had slightly higher diet NFD and HEI scores vs. foragers, though differences were not statistically significant (Figure 3). These findings indicate that while foraging behavior varies across language groups and housing conditions, its direct dietary quality impact remains unclear within this sample.

PRA Haitian Kreyòl-speaking participants listed no foraged plants. Spanish-speaking participants listed plants representing 18% of potential full PRA NFD score. Conversely, during interviews, Haitian Kreyòl speakers were more likely to forage: 54.2% participated in past-month foraging compared to 27.8% Spanish speakers and 37.9% Spanish+Indigenous speakers. However, the higher prevalence of foraging in the Haitian Kreyòl-speaking community was not statistically significant. For both Haitian Kreyòl and Spanish+Indigenous speakers, foragers reported slightly higher HEI scores. While overall combined foraging and language impact on HEI scores was not significant, language acted as a significant main effect in this model [*F*(2, 12) = 4.48, *p* = 0.02], with a large effect size (η^2^*p* = 0.23). Though larger foraging patterns among Immokalee community members remain unclear, foraging is an important source of key micronutrients (especially Vitamin A, K, and calcium) to otherwise restricted diets.

### 3.6 Nutrition resilience and diet quality: NFD vs. HEI scores

No correlation existed between NFD and HEI scores from diet recall data (*R*^2^ = 0.0058) or garden inventory data (*R*^2^ = 0.0022), suggesting that while this community maintains nutrition resilience through cultural food practices (evidenced by NFD scores), systemic barriers likely prevent available food access strategies from fully supporting optimal dietary patterns as defined by accepted nutritional standards like HEI scores.

## 4 Discussion

### 4.1 Measuring nutrition: NFD vs. HEI

This study presents an opportunity to consider methods for assessing diet in terms of both nutrition resilience (NFD) and diet quality (HEI), including examining similarities, differences, strengths, weaknesses, and opportunities of each measurement.

HEI measures dietary quality in terms of Dietary Guidelines for Americans (DGAs) alignment. The DGAs result from rigorous, multi-step scientific processes led by USDA and the Department of Health and Human Services, including scientific committee appointment, research question framing, evidence-based recommendation reports, and final guideline issuance. The DGAs consider current US population nutrition and health status, identify key concern areas, and establish recommendations addressing priority public health needs (27).

HEI operationalizes these guidelines by assigning scores based on dietary intake compared to recommended amounts. Higher HEI scores indicate eating patterns aligned with DGAs for preventing and managing chronic diseases including heart disease, type 2 diabetes, and hypertension. HEI comprises thirteen component scores: nine adequacy components (total fruits, whole fruits, total vegetables, greens and beans, whole grains, dairy, total protein, seafood and plant proteins, ratio of polyunsaturated and monounsaturated to saturated fatty acids) and four moderation components (refined grains, sodium, added sugars, saturated fats ≤ 12.2% total energy intake). These components reflect US population nutritional concern areas where average intake shows insufficient fruit and vegetable consumption alongside excessive sodium, added sugars, and saturated fat. HEI thus functions as a public health nutrition assessment tool specifically designed to measure dietary alignment with population-level guidelines addressing the most prevalent US chronic disease risks (25). However, this population-level focus raises important questions about its appropriateness for evaluating dietary quality in culturally diverse communities with distinct food traditions, access constraints, and nutritional priorities (42).

Conversely, NFD scores are summative. NFD scores reflect 34 individual nutrients, with the highest score reflecting maximum potential plant or food item aggregation available in a specific ecosystem or sample population. In this research, the highest possible NFD score encompassed all 242 plant items identified through PRA sessions and interviews. NFD is not strictly a public health measure but reflects an ecosystem, its components, and how different food plant assemblages promote system resilience. Examining nutrition from this ecological lens considers the role and contribution individual food items play in diet within broader contexts.

The observed lack of relationship between NFD and HEI suggests nutritional diversity, whether measured through consumed or grown foods, does not predict USDA dietary guideline adherence. Nutrition resilience and diet quality, as conventionally measured, appear to represent distinct food security dimensions in this population. This disconnect may hint at important Immokalee community dynamics. While between-group NFD scores demonstrate participants maintain nutrition resilience through diverse food access strategies (gardening, foraging, pantry use, exchange networks), these strategies don't translate proportionally into higher HEI scores.

Several factors may explain this observation. First, HEI is normed to US dietary guidelines that may not align with traditional Haitian, Guatemalan, and Mexican dietary patterns, potentially undervaluing culturally appropriate eating practices that nonetheless provide essential nutrients. Second, structural barriers beyond food access, including limited cooking facilities, agricultural work schedule time constraints, housing instability, and economic pressures, may prevent full utilization of available diverse food sources. Finally, nutrition resilience strategies like foraging and home gardening may function primarily as supplemental sources enhancing micronutrient intake (evidenced by elevated Vitamin A, K, and calcium from these sources) rather than serving as primary food sources shaping overall dietary patterns.

These findings underscore fundamental differences in what HEI and NFD metrics actually measure, raising important questions about applying standardized dietary guidelines across culturally diverse populations. Since HEI evaluates dietary patterns against prescriptive DGA-established recommendations, it may inadequately capture nutritional adequacy achieved through alternative food combinations or cultural dietary practices. Consider, for example, the HEI whole grain subscore. Whole grains are key DGA recommendations because they are nutrient-dense and protective against chronic disease. Their dietary pattern value stems from retaining bran and germ during processing, making them high in micronutrients like B vitamins, antioxidants, iron, magnesium, and zinc. Particularly, whole grains are high in fiber, improving digestion to reduce cholesterol, lower blood pressure, promote even blood sugars, and improve insulin sensitivity (28). Whole grains promote health and contribute importantly to diet; however, whole grains are not the exclusive means to achieve these dietary pattern goals. Brown rice, for example, may have these added benefits but also different texture and taste, potentially not preferred within certain individual or cultural eating patterns. Rice is one larger eating pattern component. Another way to achieve higher fiber and added beneficial nutrients within a diet is eating beans and white rice in combination. Rice is eaten alongside other foods, and overall presented patterns matter, not specific individual food items. Moreover, emphasizing brown rice as the “healthier” option may be unrealistic, inconsistent with individual or cultural preferences, or nutrition advice that may alienate audiences.

In contrast, NFD scores, with specific focus on macro and micronutrients, can reflect total fiber and nutrients of concern. Reported NFD score fiber may be included via brown rice, corn tortilla, vegetables, beans, or other fiber-rich foods. NFD scores may not assess diet in terms of DGA alignment but can reflect overall diet diversity as a system. Where one food item is lower in fiber, another within the larger system may provide adequate fiber. It considers diet (system) overlaps, redundancies, applications, and potential gaps as a whole. In limited food access and food insecurity contexts, considering diet as a system is meaningful, highlighting food item functions both for diet quality and potential whole system resilience.

Nutrition resilience highlights critical roles strategies like foraging play within communities like Immokalee. Foraging may not directly correlate to higher HEI scores, but within broader systems, this research implies it is a nutrition resilience tool Immokalee community members utilize within their specific food environment, reflecting their unique knowledge and expertise. This practice acts as a safety net supplementing and protecting vulnerable individual and community health. Foraging and perennial vegetable utilization resembles resilience tools highlighted in “Dutch famine” research on World War II high food scarcity periods (29). Dutch wartime diet research from 78 interviews included 14 cultivated plant species, 21 wild plant species, and 3 edible wild fungus species. In food insecurity contexts, barriers preventing healthy food access via traditional means are important to understand, but identifying barriers may not inevitably predict health outcomes as community members seek alternate solutions within food environments. As Valdez et al. (30) point out in Latino-majority Central California rural agricultural area research, communities also rely on alternate fruit and vegetable food sources including exchanges. Assessing nutrition via ecological measures like NFD helps understand foraging as resilience practice more clearly. This research project clarifies how NFD and HEI scores can be distinct but complementary measures, highlighting different food access, dignity, and community nutrition dimensions.

### 4.2 Defining foraging

Where individuals are limited in fresh fruits and vegetables, specific antioxidants or micronutrients may be lacking. Whether as preference or culture function, or as strategy to navigate land or food access lack, foraging participation increases micronutrients including antioxidants and key concern minerals. However, research participants responded to foraging questions differently in small group PRA sessions vs. individual interviews. In PRA activities, Haitian Kreyòl-speaking participants listed no foraged plants. Spanish-speaking participants listed plants representing 18% of potential full PRA session NFD score. Conversely, during interviews, Haitian Kreyòl speakers were more likely to forage: just over half participated in past-month foraging, more than Spanish and Spanish+Indigenous language speakers.

The inconsistency in reporting foraging between PRA activities and interviews may reflect unanticipated or misunderstood stigma. Neither PRA sessions nor interviews asked participants about foraging perspectives, opinions, or definitions. Helen explained that when conducting interviews, she carefully approached foraging topics to put respondents at ease. Direct Spanish “foraging” translation could imply trespassing or stealing plants. To clarify interview question intention, Helen shared childhood foraging stories as examples and asked if participants had done anything similar (H. Midney, personal communication, July 13, 2022). While many Spanish-speaking participants (both subgroups) affirmed foraging, most had not foraged within the past month compared to Haitian Kreyòl speakers.

This research cannot clarify why Haitian Kreyòl speakers didn't discuss or admit foraging in peer group settings compared to individual interviews. This distinction highlights an important point: how people understand foraging is shaped by social dynamics, individual experiences, and culture. A researcher may approach a question about foraging with a specific idea in mind—say, for example, an individual walking through a forest after a heavy rain looking for a specific species of edible mushrooms. This idea may be informed by an individual and cultural lens shaped by socioeconomic status, education, culture, and racial identity whereby someone can engage with the practice of foraging without fear of stigma, retaliation, or even threat. As Hall points out in his series of interviews with foragers, the act is both a part of cultural tradition and personal identity, and that understanding how food decisions are made and methods of procurement can be just as important as the specific food item being eaten (31). At several points during this research project, it was evident that some Immokalee community members were wary of discussing foraging for fear of stigma or safety.

Foraging reasons may differ and perhaps definitions need careful consideration to encompass broader motivation and understanding experiences. For example, foraging is not exclusive to “wild” edible plants, foraged plants listed in this research included the use of cultivated perennial vegetable species such as hoopvine. Plants foraged also includes the use of lesser used plant parts of otherwise commonly known species such as pigeon pea, orange, or catnip leaves. Perennial plants, in this way, have a multifunctional role in linking and supporting agrobiodiversity and health via ecosystem and nutrition resilience—just as Toensmeier et al., point out in their research focused on the unique value of perennial vegetables (21). Within a broader ecosystem, the role that perennial plants play to promote nutrition resilience is clear—their presence makes a source of nutritious food available during times of system vulnerability, whether that vulnerability be due to limited purchasing power, seasonal patterns, climate disturbances, insufficient transportation, or unstable housing. Perhaps further conversation is warranted to understand the links between foraging practices and perennial vegetables. Tucked away discreetly at the border of a community garden or within landscaping a perennial plant and the key nutrients it provides can be accessed when on a walk to a neighbor's home.

### 4.3 Gaps in data and knowledge

When evaluating participant-shared foraged plants during interviews, data gaps are evident. Considering these gaps more closely is paramount to understanding nutrition resilience. Composition data for several plants were unavailable through ESHA Food Processor. After researching available databases and manuscripts, several underutilized species were added to analysis. Data available for these species, such as malanga root and leaves (*Xanthosoma sagittifolium*), was less complete than plants with USDA FNDDS or USDA SR Legacy composition data, including only five of 34 nutrient values available in USDA plant data. While included, this meant several micronutrient values couldn't be added to overall NFD matrices and therefore not tabulated in NFD scores. Substitutions were also necessary. For example, only dried product nutrient composition could be used for yierba santa (*Piper auritum*), marjoram (*Origanum majorana*), and tree chili (*Capsicum anuum* “de Arbol”). Composition data lack also meant some plants were substituted with closely related species, such as Okinawa spinach (*Gynura bicolor*) for longevity spinach (*Gynura procumbens*), or Licuri (*Syagrus coronata*) for Queen palm (*Syagrus romanzoffiana*). Chilis were important foods, particularly for Spanish-speaking participants, but due to subspecies composition data lack, *Capsicum annuum* often encompassed the wide specific types list. In the end, five plants couldn't be included in the matrix. Four of these plants were foraged and often leaves of commonly known plants: orange leaves (*Citrus x sinensis*), catnip leaves (*Nepeta cataria*), pigeon pea leaves (*Cajanus cajan*), and jatropha seeds (*Jatropha curcas*).

Within foraged plant lists, one species particularly merits note. *Trichostigma octandrum* was both gardened and foraged, consumed by several research participants. Commonly known as Haitian basket vine or hoopvine, it is a perennial vegetable listed several times during community PRA sessions. Due to nutrition composition data lack, hoopvine couldn't be tabulated during PRA NFD analysis, diet analysis, or garden analysis. Hoopvine absence illustrates Beltrame et al.'s (32) points well: where composition data is missing, plants are unreported. When unreported, they're not included in analysis, and their nutritional functional diversity contribution to greater food systems is overlooked. In turn, community workers may not have opportunities to become familiar with hoopvine. Next, educators don't share or promote potential hoopvine uses and benefits. One begins seeing downstream realities where policy and markets more likely promote regional food system uniformity and in turn increase vulnerability. Relying on generic composition data doesn't adequately reflect individual food plant functions within broader food dietary ecosystems (33). As Burlingame et al. (34) argue, composition data for species level and below is needed to make links between agroecology and nutrition, strengthen interdisciplinary cooperation and potential interventions, and promote biodiversity. Within nutrition science, the cost of not understanding foraging definition nuance and plants sourced via foraging overlooks valuable nourishment sources and plants valued by individuals or communities, simultaneously missing opportunities to understand and support nutrition resilience. Harm is caused when community members and their expertise are marginalized this way.

### 4.4 Defining nutrition education and exchange

It appears that our research participants had access to diverse and consistent sources of produce and nutrition from other means than just gardens. In fact, individuals renting rooms with limited land access still reported highest diet NFD scores. One study weakness is its cross-sectional nature, assessing preferred plants, home gardens, and diet at one time snapshot. It didn't consider Cultivate Abundance work as intervention within the Immokalee community's broader food landscape where food access barriers are evident. Fresh fruit and vegetable item presence in food pantries via Cultivate Abundance network represents not just plant diet diversity and key concern nutrient access, but items familiar to community members from pre-migration settings, including sometimes preferred food plants otherwise listed as difficult to access. The opportunity to offer these specific plant species is impacted by Southwest Florida's unique subtropical environment (hardiness zone 10a), which allows many Haitian and Central American food heritage-associated crops to be more readily grown compared to colder continental United States locations. The PRA session indicated that plants most difficult to access had the highest %NFD scores, and that those accessed by way of food pantry had the second highest %NFD scores. This may indicate that the presence of fruits and vegetables via Cultivate Abundance in the Misión Peniel food pantry is an access point that improves diet diversity and supports health of pantry participants. As a result of this work, overall diet diversity of research participants may have, in part, reflected the produce made available to them through Cultivate Abundance's programs, along with other sources. Nutrition resilience, as a specific dimension of food system resilience, considers both individual and institutional actors (2). Cultivate Abundance, as a nonprofit actor, shapes nutrition resilience in the way it supports and manages activities within Immokalee's food system.

Cultivate Abundance's work centers on three activities: growing, collecting, and sharing. What's important to note is how this process includes sharing not just food plants but knowledge as well. Shared knowledge includes both plant species and food preferences. The 33 home gardens contributing to produce available on Misión Peniel pantry days include gardens in Lee and Collier counties, including Fort Myers, Naples, and within Immokalee. In fact, the same participants attending pantry distributions are also connected to Cultivate Abundance as produce donors. In food pantry lines, Cultivate Abundance staff may offer leafy greens such as Hierba (yierba) mora (*Solanum americanum*), a short-lived perennial plant. Its food pantry incorporation reflects neighbor conversations. Rick Burnette recalls a Haitian farmworker entering the food pantry line with an armful of Hierba mora seedlings, likely foraged from agricultural fields while working. When other neighbors in food pantry lines showed interest in plants, the seedling-carrying neighbor shared them. Rick shared that he watched this exchange repeat when another Immokalee community member showed up with new seedling batches (R. Burnette, personal communication, October 2023). Observing this exchange echoes findings from Vorstenbosch et al.'s (29) ethnobiology work focused on Dutch famine foraging. While the Dutch government sought to increase wild edible plant knowledge during wartime cookbooks, most information about plants like tulip bulbs and sugar beets was shared via family members and neighbors by word of mouth. By being surrounded by wild foods and hearing stories, people may become more familiar with these plants and more likely to collect and consume them. In Immokalee, some farmworkers‘ unique knowledge meant they were familiar with Hierba mora value and culinary use, sharing it with others. In noting this value, Cultivate Abundance can participate in food plant conversation and exchange, center their importance, and emphasize not just their potential dietary use but Immokalee neighbors' contributions and expertise.

The presence of plants like Hierba mora in the Misión Peniel represents a form of nutrition education that subverts a more hierarchical exchange between “teacher” and “student.” This subversion is consistent with what Rahman et al. (35) refer to as “co-creation of knowledge” in “The Social Ecology of Food: Where Agroecology and Heritage Meet”. Traditional public health nutrition education prioritizes nutrients of concern and designs interventions to promote population health. Where most Americans do not consume the recommended amount of daily fruits and vegetables, messaging emphasizing the importance of fruits and vegetables is prioritized. A nutrition education curriculum is written to highlight the value of fruits and vegetables and techniques to incorporate them into daily eating patterns. Public health nutrition programs aimed to support children via school food, older adults via congregate and non congregate feeding programs, and food insecure communities via The Emergency Food Assistance Program are required to meet recommended fruit and vegetable intake. Messaging is grounded in science, but designed as a top-down intervention. Where can public health nutrition programs be designed as more responsive to the unique context and skillset of the individual consumer? Co-creation of knowledge not only supports more relevant public health nutrition messaging, but prioritizes local food knowledge as a foundation for sustainable food systems change (35). Knowledge co-creation is a complex, but strategic steps can include participatory approaches to community programs, including elder generations in conversations, hosting nature walks with knowledgeable neighbors, and protecting the right to forage plants in community green spaces (36).

Nutrition education is not limited to a classroom or farmers market cooking demonstration. It also happens in the food pantry line, in conversations about a soup prepared the day before, in sharing a story about a memory of a meal prepared by a caregiver, or even asking someone to taste a vegetable leaf and share their opinion. It happens when a neighbor carries leaves foraged from a field and one individual observes another becoming excited about the plant. In valuing these exchanges, recognizing their importance, and reinforcing their value, communities create meaning. That meaning is shared via garden networks that appreciate, promote, and share plants that have different unique functions—some that are for celebratory dishes, some for their easy reliability to grow, some that offer specific marked flavor, some that offer versatility in the parts that can be used for different meals, and others for their ability to be easily transplanted or grown in small spaces. A nutrition functional diversity lens helps one to see the ways that each plant is a tool for broader nutrition resilience. Understanding the role that each method plays and why each plant is important is one way that communities communicate to others not just plant food knowledge but also the depth and strength of their own expertise. When community nutrition programs fail to see this exchange as a form of reciprocity and education, opportunities missed but harm can be caused. When community nutrition programs pause to understand these exchanges as a form of education (between neighbors, generations, across cultures), nutrition resilience is strengthened.

In the context of structural issues related to food access, economics, migration, and social determinants of health, community members in Immokalee, Florida make choices around food procurement and consumption for a myriad of reasons. These choices made are consistent with the ways that Zurek et al. (37), describe food system resilience as encompassing actors' to resist disruption, capacity to recover from shock, and their subsequent reorientation. They write, “Actors will not adapt their activities without reason, but in response to a changed driver(s) to either capitalize on an opportunity or mitigate a threat.” Participants in this study evidently carry strategies for nutrition resilience. In “Not yet at the table: The absence of food culture and tradition in agroecology literature,” the authors argue that academic work around agroecology mentions cultural dynamics in passing, and with the assumption that promoting agroecology will naturally result in unspecified healthy, diversified, and community-driven food choices, but little critical work has been done to explore these connections (38). Understanding the links requires a deeper knowledge and conversation around foodways that are embodied in kitchen spaces and food pantry lines. Food culture is forever changing, it is complex and shaped by many factors. Decisions made around food may also be rooted in relationships, social capital, dignity, preference, therapeutic elements, harm reduction, identity, etc. (40). This research reflects a part of those complexities, informs questions, and illuminates the need for cultural humility in seeking answers. There are opportunities at hand to learn from the culture and strategies for nutrition resilience that farmworkers carry with them. In doing so, dietitians and healthcare professionals may be better equipped to improve upstream issues that shape health outcomes within the communities within which they work.

## 5 Conclusion

Our findings underscore that interventions to improve food security must address both nutritionally diverse food availability and systemic conditions enabling their consumption in health-supporting patterns. Farmworker community members in Immokalee, Florida face structural barriers as a form of everyday violence, including difficulty in accessing food. Barriers to access include threat of immigration and customs enforcement agents, lack of transportation, low earning power combined with high cost of food, and long distances to travel to grocery stores located outside of the community. In this context, individuals use their unique experiences and preferences to source food. Strategies include gardening, food pantries, exchange, and foraging. Food items ranked as those most difficult to access in the PRA session had the highest %NFD score, just above those accessed via the Misión Penial food pantry.

Farmworker community members in Immokalee, Florida hold nutrition resilience strategies, practices evident in subtle ways. Responses to foraging questions and conversation varied based on language used and topic framing, including what plants and foraging meant to people based on their own experiences. It was important to be careful to reduce potential foraging-associated stigma. While foraging didn't necessarily improve HEI or NFD scores, it did provide access to key micronutrients of concern in otherwise restricted food environments. Foraging is one way that research participants pull in perennial vegetables from the broader ecosystem as a tool for nutrition resilience, a reality captured more readily by NFD scores. An NFD measure may not correlate with improved HEI measures, but it offers a different perspective on diet quality where individual dietary choices are considered for their function and role within a larger system and its capacity to respond to and recover from shock. By way of next steps, longitudinal studies or a controlled study evaluating participants NFD and HEI scores prior to and after participating in the Misiòn Peniel pantry, and throughout the annual calendar, would clarify the relationship between diet, foraging, and garden diversity. Similarly, Cultivate Abundance may consider compiling an Immokalee plant database interviewing participants' understanding and use of diverse plants while interacting with and exchanging knowledge around these food plants with neighbors through the Misiòn Peniel pantry and garden over time. Knowledge about food plants and foraging is shared between community members through day-to-day interactions. When community and local government organizations participate in that exchange as learners and co-collaborators, the care provided - whether it be in growing, sharing, and distributing food and information about nutrition - the quality and relevancy of programs and interventions improve.

## Data Availability

The datasets presented in this article are not readily available due to ethical approval and participant confidentiality protection. Requests to access the datasets should be directed to the corresponding author.
